# White matter microstructure alterations in type 2 diabetes mellitus and its correlation with cerebral small vessel disease and cognitive performance

**DOI:** 10.1038/s41598-023-50768-z

**Published:** 2024-01-02

**Authors:** Yangyingqiu Liu, Yuhan Jiang, Wei Du, Bingbing Gao, Jie Gao, Shuai Hu, Qingwei Song, Weiwei Wang, Yanwei Miao

**Affiliations:** 1 Department of Radiology, First Affiliated Hospital of Dalian Medical University, 222 Zhongshan Road, Xigang, Dalian, China; 2 Department of Radiology, Zibo Central Hospital, 54 Gongqingtuan Road, Zhangdian, Zibo, China; 3 Department of Neurology, First Affiliated Hospital of Dalian Medical University, 222 Zhongshan Road, Xigang, Dalian, China

**Keywords:** Cognitive neuroscience, Type 2 diabetes

## Abstract

Microstructural abnormalities of white matter fiber tracts are considered as one of the etiology of diabetes-induced neurological disorders. We explored the cerebral white matter microstructure alteration accurately, and to analyze its correlation between cerebral small vessel disease (CSVD) burden and cognitive performance in type 2 diabetes mellitus (T2DM). The clinical-laboratory data, cognitive scores [including mini-mental state examination (MMSE), Montreal cognitive assessment (MoCA), California verbal learning test (CVLT), and symbol digit modalities test (SDMT)], CSVD burden scores of the T2DM group (n = 34) and healthy control (HC) group (n = 21) were collected prospectively. Automatic fiber quantification (AFQ) was applied to generate bundle profiles along primary white matter fiber tracts. Diffusion tensor images (DTI) metrics and 100 nodes of white matter fiber tracts between groups were compared. Multiple regression analysis was used to analyze the relationship between DTI metrics and cognitive scores and CSVD burden scores. For fiber-wise and node-wise, DTI metrics in some commissural and association fibers were increased in T2DM. Some white matter fiber tracts DTI metrics were independent predictors of cognitive scores and CSVD burden scores. White matter fiber tracts damage in patients with T2DM may be characterized in specific location, especially commissural and association fibers. Aberrational specific white matter fiber tracts are associated with visuospatial function and CSVD burden.

## Introduction

In patients with type 2 diabetes mellitus (T2DM), cognitive impairments tend to affect verbal and visual memory, information processing speed, and executive functioning^1^, particularly among people above 65 years^2^. Cerebral vascular injury is one of the possible mechanisms for the increased risk of cognitive impairment in T2DM patients^3,4^, especially microvascular. In addition to the classic target organs of microvascular complications such as the retina and kidney, the brain is also considered as the target organ of microvascular complication in patients with T2DM^5^.

Cerebral small vessel disease (CSVD) is a neuroradiological diagnosis that refers to a group of pathological processes affecting small arteries, arterioles, capillaries, and small veins in the brain^6^. Cerebral small vessels form a small vessel network, which is embedded in the neurovascular unit (NVU) and participates in the formation of the blood–brain barrier, regulating substances from the blood to the brain parenchyma, regulating cerebral blood flow (CBF), maintaining and repairing myelin sheath, clearing metabolites, and other functions. NVU is linked to neurodegenerative diseases and cognitive impairments^7^.

Individuals with T2DM exhibit a higher prevalence of CSVD in comparison with the general population^8–10^. CSVD may be one of the key underlying mechanisms of cognitive dysfunction in patients with T2DM^11^. However, most small cerebral vessels are difficult to be visualized directly in vivo. The development of magnetic resonance imaging (MRI) has made it possible to identify and detect CSVD in vivo and non-invasively. According to the Standards for Reporting Vascular changes on Neuroimaging-2 (STRIVE-2), MRI findings including recent small subcortical infarct (RSSI), lacune, white matter hyperintensity (WMH), perivascular space (PVS), cerebral microbleed (CMB), cortical superficial siderosis, cortical microinfarct, and atrophy have been used as markers for CSVD^12^.

In recent years, studies generally believe that white matter fiber tracts are damaged in T2DM patients^13–15^, and related to the metabolic disorder^16^, microvascular dysfunction, and decreased cognitive function^17^. Commissural, projection and association fibers are the three major classes of white matter fiber tracts^15^. Microstructural abnormalities of these white matter fiber tracts are an important biomarker, and considered as the etiology of diabetes-induced neurological disorders^18^. Diffusion tensor images (DTI) is improved, developed, and evolved based on diffusion weighted imaging (DWI). It can display brain white matter fiber tracts non-invasive and can qualitatively and quantitatively evaluate the microstructure changes of the white matter. Still, most studies use processing methods based on regions of interest, voxel-based analysis (VBA), tract-based spatial statistics (TBSS), etc. However, these methods have problems such as the limitation of analysis scope, and individual levels cannot be positioned accurately. As an automatic quantitative analysis technology of diffusion tensor images (DTI) tractography technique, automated fiber quantification (AFQ) overcomes the shortcomings of VBA and TBSS. It can automatically extract 20 primary white matter fiber tracts of the whole brain, analyze the diffusion indicators along the length of white matter fiber tracts, and divide each fiber into 100 equidistant nodes for node analysis^19^. AFQ is more sensitive to the alterations in white matter microstructure and achieve the accurate positioning of white matter fiber tracts. AFQ can show abnormal changes of some segments without obvious changes of the whole fiber. AFQ has been applied to study white matter microstructure changes in Alzheimer disease, epilepsy, and end-stage renal disease encephalopathy^20–24^.

In the present study, we hypothesize that white matter damage may vary along fiber tracts in patients with T2DM and may provide potential biomarkers for the cognitive performance and CSVD burden. We attempt to investigate the CSVD burden and white matter fiber tracts alterations in T2DM patients, locate abnormal fiber nodes accurately and analyze the relationship between white matter DTI metrics and cognitive performance and CSVD burden.

## Materials and methods

### Participants

This prospective study was approved by the First Affiliated Hospital of Dalian Medical University Institutional Review Board (No. PJ-KS-KY-2021-121, July 3, 2021). All participants gave written informed consent before participation. This study was conducted in accordance with the principles of the Declaration of Helsinki.

The inclusion criteria for T2DM group were: (1) clinical diagnostic criteria of T2DM recommended by the American Diabetes Association (ADA) in 2014^25^; (2) right handed, handedness is a behavioral reflection of functional lateralization, and different habitual hands have different processing methods in the field of neurocognition^26^; (3) no contraindication of MRI scan. Exclusion criteria included: (1) serious medical emergency (severe anemia, heart failure, renal failure, severe electrolyte disorders, etc.); (2) history of drug and poison abuse or dependence, alcoholism, etc.; (3) current or previous history or family history of neuropsychiatric diseases (schizophrenia, mania, depression, etc.); (4) severe brain disease (cerebral hemorrhage, massive cerebral infarction, drug-induced encephalopathy, malignant tumors, etc.); (5) poor MRI image quality.

Inclusion criteria for HC group were: (1) gender, age, race and education were matched with T2DM patients; (2) right handed; (3) previous health; (4) no contraindication of MRI scan. Exclusion criteria were: (1) history of diabetes and hypertension; (2) brain MRI examination showed abnormal development of brain structure; (3) the other exclusion criteria were the same as those of T2DM group (items 1–5).

Finally, a total of 55 participants were recruited, 34 T2DM patients composed the T2DM group, and 21 healthy controls (HC) composed the HC group.

### Clinical and laboratory data

Clinical data were collected for all participants, including gender, age, height, weight, waist circumference, hip circumference, systolic blood pressure (SBP), diastolic blood pressure (DBP), education years, body mass index (BMI) and waist to hip ratio (WHR). All participants underwent several laboratory tests, including fasting glucose (FG), glycated hemoglobin (HbA1c), total cholesterol (TCHOL), triglyceride (TG), low-density lipoprotein (LDL), high-density lipoprotein (HDL), homocysteineacid (HCY) within one week before MR data acquisition.

### Neurocognitive assessments

All participants were evaluated by a neuropsychological physician (J.G.) with a detailed neuropsychological scale, including mini-mental state examination (MMSE), Montreal cognitive assessment (MoCA), California verbal learning test (CVLT), and symbol digit modalities test (SDMT). MMSE used to evaluates global cognitive function, including orientation, attention, calculation, executive function, language, among others^27^. MoCA mainly used for a brief evaluation of cognitive functions such as memory, visuospatial, abstract thinking, attention, and executive function^28^, it is more sensitive than MMSE for detection of mild cognitive impairment in T2DM^29^. Verbal episodic memory was measured by CVLT, measuring verbal auditory learning, recall- and recognition memory^30^. SDMT was used for evaluating execution functions^31^. All tests were performed before the MRI scan on the same day.

### MRI acquisition

All participants were imaged using a 3.0 T MRI scanner (Ingenia CX, Philips Healthcare, Best, the Netherlands) equipped with a 32-channel phased-array head coil. High-resolution, three-dimensional (3D), T1-weighted (T1W) images were obtained using a multi-shot turbo field echo (MSTFE) sequence with the following scan parameters: echo time (TE) = 3.0 ms, repetition time (TR) = 6.6 ms, flip angle = 12°, slices = 188, field of view (FOV) = 256 × 256 mm^2^, matrixsize = 256 × 256, and thickness = 1.0 mm. We obtained the DTI data using a single-shot echo planar imaging (SSEPI) sequence (TE = 92 ms, TR = 6000 ms, FA = 90°, voxel size = 2 × 2 × 2 mm^3^, FOV = 256 × 256 mm, matrix size = 128 × 128, 68 axial slices of 2 mm thickness to cover the whole brain without gap). Each DTI dataset included 64 noncollinear spatial directions at b-value = 1000 s/mm^2^ and one baseline image at b = 0 s/mm^2^.

### CSVD scores

Two radiologists with 10 years and 6 years of neuroradiology experience (Y.L., Y.J.) provided the CSVD scores of all participants independently, including WMH grade, EPVS score, lacune number, CMB number, and CSVD total burden score. The detailed definition and criteria for WMH grade, EPVS score, and CSVD total burden score were shown in the Supplementary materials.

### Data preprocessing

DTI was preprocessed by the Vistasoft package (version 1.0) (https://github.com/vistalab/vistasoft). Functional MRI of the Brain (FMRIB) Software Library (FSL) (version 5.0.9^2^) (http://fsl.fmrib.ox.ac.uk/fsl/) was used to correcteddy current-induced distortion, motion artifact, strip skull, and generate the following diffusion metrics values: fractional anisotropy (FA), mean diffusivity (MD), λ1, λ2, λ3, axial diffusivity (AD), radial diffusivity (RD). Brain Extraction Tool (BET) in FSL was used to remove non-brain structures for 3D T1WI scans, then images were averaged and rotated to align with the anterior–posterior commissure plane. The diffusion metrics along the fibers were quantified by applying AFQ^19^. The 20 primary fibers were preliminarily determined, including bilateral corticospinal tract (CT), bilateral cingulum cingulated (CC), bilateral inferior fronto-occipital fasciculus (IFOF), bilateral thalamic radiation (TR), bilateral inferior longitudinal fasciculus (ILF), bilateral superior longitudinal fasciculus (SLF), bilateral uncinate, callosum forceps Major (CF_ Major), callosum forceps Minor (CF_ Minor), bilateral arcuate fasciculus (AF) and bilateral cingulum hippocampus (CH). The diffusion metrics of 100 equidistant nodes on each fiber were measured. However, due to the strict criterion for tract segmentation, we failed to identify bilateral AF and CH. Finally, 16 fibers were completely tracked and identified.

### Statistical analyses

#### Sample size calculation

The sample size was calculated using Power Analysis and Sample size (PASS) software version 15.0.5. The calculation was done based on two-tailed test, α of 0.05, and power of 0.8, the ratio of sample size between T2DM group and HC group was 1:1. Referring to previous studies^15,32–34^, the diffusion metrics values of bilateral IFOF, CF_Major, CF_Minor were used as the main observation indicators. The data of our preliminary experiment were used for sample size calculation.

#### Clinical data, laboratory data, cognitive scores, and CSVD scores

Data analyses were performed using the Statistical Package for Social Science (SPSS) version 22.0. Independent samples t-test or Mann–Whitney U test was used to compare the difference in measurement data between T2DM and HC group. Chi-square test was used to compare the difference in enumeration data between T2DM and HC groups. *p* < 0.05 was considered statistically significant.

#### Diffusion metric

The mean values of FA, MD, AD, and RD of 16 fibers was compared betweenT2DM and HC groups. Controlling gender, age, BMI, and education as covariates, node-wise analyses were applied based on the general linear model (GLM) permutation test (5000 permutations) in the FSL to compare the differences in 100 nodes diffusion metrics of 16 fibers between the T2DM group and the HC group. Only significant differences observed at ≥ 3 adjacent nodes were reported^35^.

We then performed multiple linear regression analysis to find independent predictors for explaining the relationship between DTI metrics and cognitive and CSVD scores, age, gender, education years were inserted as control variables^36^. Statistical tests were two-tailed, and *p* < 0.05 was considered statistically significant.

## Results

### Sample size calculation

The estimated sample size was at least 40. While the total sample size in our study was 55 participants. Therefore, the total sample size for this study was sufficient to meet the accuracy requirements of the study. The details of sample size calculation were shown in Supplementary Table S1.

### Clinical and laboratory data

The clinical and laboratory data of T2DM and HC groups were summarized in Table 1. Age, gender, education years, DBP, TCHOL, LDL, and HCY between groups showed no statistical difference (*p* > 0.05). The SBP, BMI, WHR, FG, HbA1C and TG were increased, and the HDL was decreased in T2DM patients compared with HC group (*p* < 0.05).

**Table 1:** Clinical and laboratorydata of T2DM and HC groups.

	HC group (n = 21)	T2DM group (n = 34)	Statistical value (χ^2^/t/Z)	*p* value
Gender (male), n (%)	7 (33.33%)	18 (52.94%)	2.013	0.177
Age (years)^a^	56.381 ± 7.768	60.677 ± 9.591	− 1.730	0.089
Number aged 40–49, n (%)	5 (23.810%)	6 (17.647%)	3.371	0.185
Number aged 50–59, n (%)	6 (28.571%)	4 (11.765%)		
Number aged 60 and above, n (%)	10 (47.619%)	24 (70.588%)		
Education years^b^	12.000 (6.000)	12.000 (3.000)	− 1.600	0.110
SBP (mmHg)^b^	120.000 (10.000)	130.000 (19.250)	− 3.266	0.001*
DBP (mmHg)^b^	78.000 (13.000)	80.000 (10.750)	− 1.644	0.100
BMI^a^	22.937 ± 2.736	24.899 ± 2.260	− 2.884	0.006*
WHR^b^	0.904 (0.075)	0.926 (0.079)	− 2.651	0.008*
FG (mmol/L)^b^	4.740 (0.520)	7.640 (3.440)	− 5.582	< 0.001*
HbA1c (%)^a^	5.495 ± 0.291	7.597 ± 1.373	− 6.899	< 0.001*
TChol (mmol/L)^a^	5.028 ± 1.041	4.802 ± 1.016	0.793	0.431
TG (mmol/L)^b^	1.180 (0.740)	1.485 (1.040)	− 2.088	0.037*
LDL (mmol/L)^a^	2.724 ± 0.719	2.602 ± 0.648	0.650	0.519
HDL (mmol/L)^a^	1.293 ± 0.281	1.076 ± 0.257	2.930	0.005*
HCY (mmol/L)^b^	10.950 (5.170)	12.230 (4.355)	− 0.208	0.835

*T2DM* type 2 diabetes mellitus; *HC* healthy control; *SBP* systolic blood pressure; *DBP* systolic blood pressure; *BMI* body mass index; *WHR* waist to hip ratio; *FG* fasting glucose; *HbA1c* glycated hemoglobin; *TChol* total cholesterol; *TG* triglyceride; *LDL* low-density lipoprotein; *HDL* high-density lipoprotein; *HCY* homocysteineacid.

*p < 0.05.

^a^data are expressed as mean value ± standard deviations.

^b^data are expressed as median (inter-quartile range).

### Cognitive scores

The cognitive scores of T2DM and HC groups were shown in Table 2. The total MMSE score, MoCA visuospatial score, total MoCA score, and SDMT score were decreased in T2DM group compared with HC group (*p* < 0.05).

**Table 2:** Cognitive scores of T2DM and HC groups.

		HC group (n = 21)	T2DM group (n = 34)	Statistical value (χ^2^/t/Z)	*p* value (FDR corrected)
MMSE	orientation^b^	10.00 (0)	10.00 (0)	0	1.000
	immediate recall^b^	3.00 (0)	3.00 (0)	0	1.000
	attention and calculation^b^	5.00 (0)	5.00 (0.75)	− 2.051	0.207
	delay recall^b^	2.00 (2.00)	2.00 (2.00)	− 1.075	0.585
	Language (naming)^b^	2.00 (0)	2.00 (0)	− 0.786	0.670
	Language (repetition)^b^	1.00 (0)	1.00 (0)	− 0.786	0.670
	Language (reading)^b^	1.00 (0)	1.00 (0)	0	1.000
	Language (command)^b^	3.00 (0)	3.00 (0)	0	1.000
	Language (writing)^b^	1.00 (0)	1.00 (0)	− 1.617	0.393
	construction^b^	1.00 (0)	1.00 (0)	− 0.870	0.658
	Total MMSE^b^	29.00 (2.00)	28.00 (2.75)	− 2.049	0.047*
MoCA	visuospatial^b^	2.00 (1.50)	1.00 (1.00)	− 2.749	0.047*
	draw clock^b^	3.00 (0)	3.00 (0.75)	− 0.997	0.618
	naming^b^	3.00 (0)	3.00 (0)	0	1.000
	attention^b^	6.00 (0)	6.00 (0)	− 1.172	0.534
	language^b^	3.00 (0)	3.00 (0.75)	− 1.580	0.393
	abstraction^b^	2.00 (0)	2.00 (0)	0	1.000
	delayed recall^b^	3.00 (1.50)	2.00 (1.00)	− 2.077	0.207
	orientation^b^	6.00 (0)	6.00 (0)	0	1.000
	Total MoCA^a^	27.143 ± 1.424	25.353 ± 2.241	3.621	0.031*
CVLT	T1 free recall correct^b^	5.00 (1.00)	5.00 (2.75)	− 0.062	1.000
	T2 free recall correct^a^	7.380 ± 2.334	7.294 ± 1.977	0.148	1.000
	T3 free recall correct^a^	9.190 ± 1.721	8.765 ± 2.463	0.693	0.692
	T4 free recall correct^a^	10.143 ± 1.905	9.294 ± 2.355	1.392	0.527
	T5 free recall correct^a^	10.857 ± 2.476	9.824 ± 1.850	1.767	0.368
	Total free recall correct^a^	42.524 ± 6.925	39.912 ± 8.451	1.190	0.534
	SDFR^b^	9.000 (2.000)	9.000 (2.000)	− 1.243	0.534
	SDCR^a^	10.286 ± 1.821	9.794 ± 2.115	0.882	0.658
	LDFR^b^	9.000 (3.000)	9.000 (2.000)	− 0.719	0.692
	LDCR^a^	10.381 ± 2.397	9.500 ± 2.711	1.222	0.534
SDMT	SDMT^b^	52.000 (25.000)	34.000 (23.750)	− 2.792	0.047*

*T2DM* type 2 diabetes mellitus; *HC* healthy control; *MMSE* mini-mental state examination; *MoCA* Montreal cognitive assessment; *CVLT* California verbal learning test; *SDFR* short delay free recall; *SDCR* short delay cued recall; *LDFR* long delay free recall; *LDCR* long delayed cued recall; *SDMT* symbol digit modalities test.

*p < 0.05.

^a^data are expressed as mean value ± standard deviations.

^b^data are expressed as median (inter-quartile range).

### CSVD scores

The CSVD scores of T2DM and HC groups were shown in Table 3. The PVH grade, DWMH grade, CSO-EPVS score, BG-EPVS score, and CSVD total burden score of T2DM group were higher than HC group (*p* < 0.05).

**Table 3:** CSVD scores of T2DM and HC groups.

			HC group (n = 21)	T2DM group (n = 34)	Statistical value (χ^2^/t/Z)	*p* value
Fazakas WMH grade	PVH	0	2 (9.524%)	0	12.86	0.005*
		1	15 (71.429%)	13 (38.236%)		
		2	3 (14.286%)	19 (55.882%)		
		3	1 (4.762%)	2 (5.882%)		
	DWMH	0	3 (14.286%)	0	13.30	0.004*
		1	14 (66.667%)	13 (38.236%)		
		2	3 (14.286%)	14 (41.176%)		
		3	1 (4.762%)	7 (20.588%)		
EPVS score	CSO	0	0	0		< 0.001*
		1	15 (71.429%)	1 (2.940%)	35.27	
		2	5 (23.810%)	10 (29.412%)		
		3	1 (4.762%)	20 (58.824%)		
		4	0	3 (8.824%)		
	BG	0	0	0		< 0.001*
		1	14 (66.667%)	1 (2.941%)	29.70	
		2	6 (28.571%)	22 (64.706%)		
		3	1 (4.762%)	11 (32.353%)		
		4	0	0		
Lacune number		–	0 (0)	0 (0)	− 0.584	0.559
CMB number		–	0 (0)	0 (1.00)	− 1.024	0.306
CSVD total burden score		0	12 (57.143%)	3 (8.824%)	20.57	< 0.001*
		1	6 (28.571%)	8 (23.539%)		
		2	1 (4.762%)	12 (35.294%)		
		3	1 (4.762%)	7 (20.588%)		
		4	1 (4.762%)	4 (11.765%)		

*CSVD* cerebral small vessel disease; *T2DM* type 2 diabetes mellitus; *HC* healthy control; *WMH* white matter hyperintensity; *PVH* periventricular hyperintensity; *DWMH* deep white matter hyperintensity; *EPVS* enlarged perivascular space; *CSO* central semi oval; *BG* basal ganglia; *CMB* cerebral microbleed.

*p < 0.05.

^a^data are expressed as mean value ± standard deviations.

^b^data are expressed as median (inter-quartile range).

### Diffusion metric

**Figure 1:**
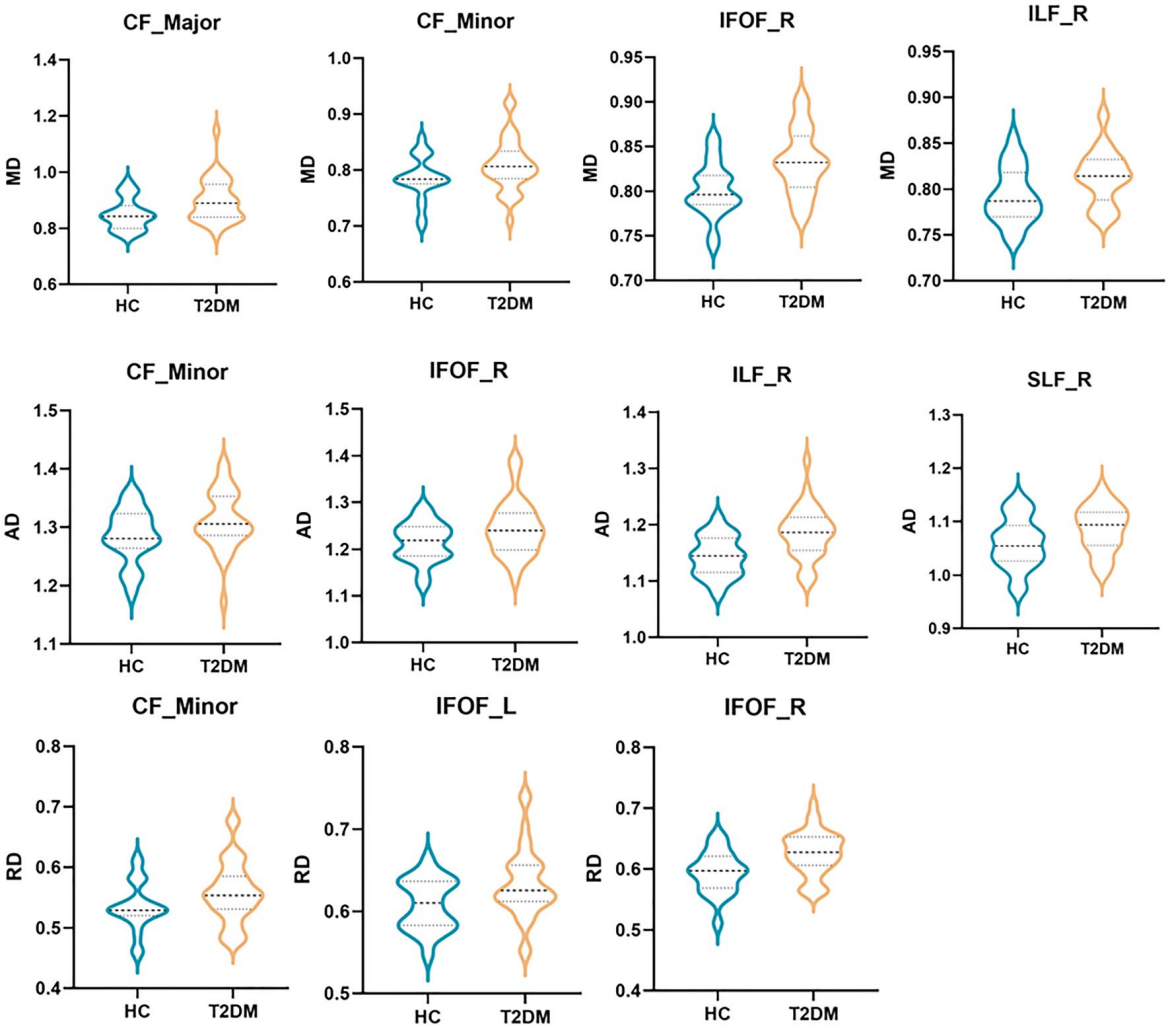
Fibers with statistical differences between T2DM and HC group. *T2DM* type 2 diabetes mellitus; *HC* healthy control; *MD* mean diffusivity; *AD* axial diffusivity; *RD* radial diffusivity; *CF* callosum forceps; *IFOF* inferior fronto-occipital fasciculus; *ILF* inferior longitudinal fasciculus; *SLF* superior longitudinal fasciculus; *R* right; *L* left*.*

**Figure 2:**
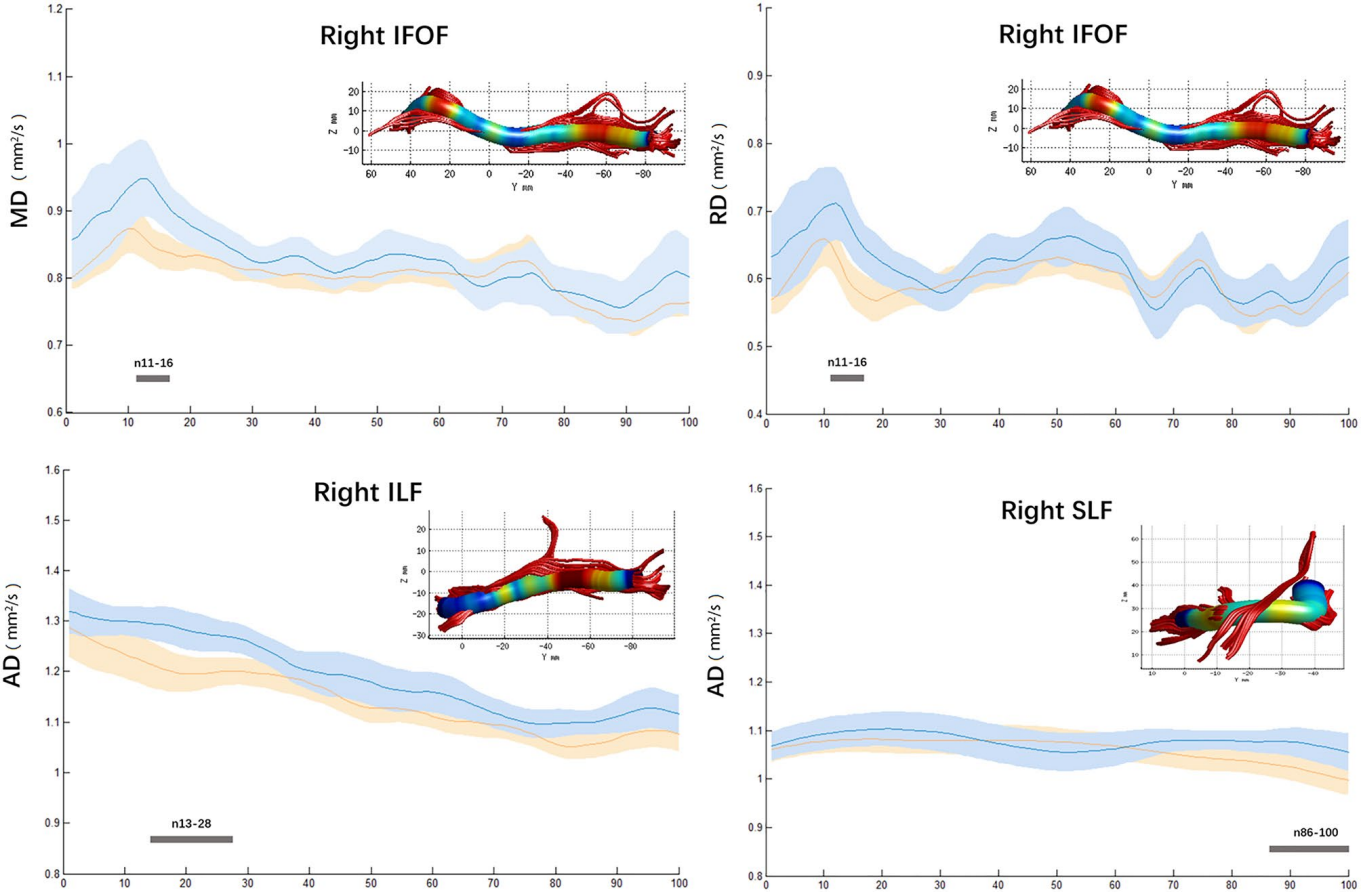
Plots of significantly altered locations in point-wise comparison between T2DM and HC group (*p* < 0.05, FWE corrected). The orange and blue lines represent the HC and T2DM groups; respectively (solid lines for mean value and shaded regions for confidence interval). The pink bar represents fiber nodes with significant differences between the two groups. *T2DM* type 2 diabetes mellitus; *HC* healthy control; *MD* mean diffusivity; *AD* axial diffusivity; *RD* radial diffusivity; *IFOF* inferior fronto-occipital fasciculus; *ILF* inferior longitudinal fasciculus; *SLF* superior longitudinal fasciculus.

For fiber-wise, the mean MD value in CF_Major, CF_Minor, right IFOF, right ILF, the mean value of AD in CF_Major, right IFOF, right ILF, right SLF, the mean RD value of RD in CF_Major, bilateral IFOF, were increased in T2DM compared to HC group (Table 4, Fig. 1). The nodes 11–16 of MD and RD in right IFOF, nodes 86–100 of AD in right SLF, and nodes 13–28 of AD in right ILF in T2DM patients were increased in the node-wise comparison between groups (Fig. 2) (FWE corrected).

**Table 4:** Diffusion metrics of T2DM and HCgroups.

		HC group (n = 21)	T2DM group (n = 34)	Statistical value (χ2/t/Z)	*p* value (FWE corrected)
MD	CF_Major^a^	0.849 ± 0.055	0.897 ± 0.075	− 2.530	0.031
	CF_Minor^a^	0.784 (0.022)	0.806 (0.045)	− 2.044	0.045
	IFOF_R^a^	0.801 ± 0.030	0.832 ± 0.036	− 3.303	0.007
	ILF_R^a^	0.793 ± 0.030	0.813 ± 0.0296	− 2.488	0.031
AD	CF_Major^a^	1.515 ± 0.055	1.558 ± 0.075	− 2.311	0.034
	IFOF_R^a^	1.215 ± 0.043	1.245 ± 0.059	− 2.027	0.048
	ILF_R^a^	1.144 ± 0.039	1.185 ± 0.045	− 3.527	0.007
	SLF_R^a^	1.060 ± 0.047	1.087 ± 0.043	− 2.150	0.044
RD	CF_Major^a^	0.517 ± 0.061	0.567 ± 0.082	− 2.426	0.031
	IFOF_L^a^	0.608 ± 0.031	0.634 ± 0.043	− 2.405	0.031
	IFOF_R^a^	0.594 ± 0.035	0.626 ± 0.036	− 3.229	0.007

*T2DM* type 2 diabetes mellitus; *HC* healthy control; *MD* mean diffusivity; *AD* axial diffusivity; *RD* radial diffusivity; *CF* callosum forceps; *IFOF* inferior fronto-occipital fasciculus; *ILF* inferior longitudinal fasciculus; *SLF* superior longitudinal fasciculus; *R* right; *L* left.

*p < 0.05.

^a^data are expressed as mean value ± standard deviations.

### Multiple linear regression analysis

**Figure 3:**
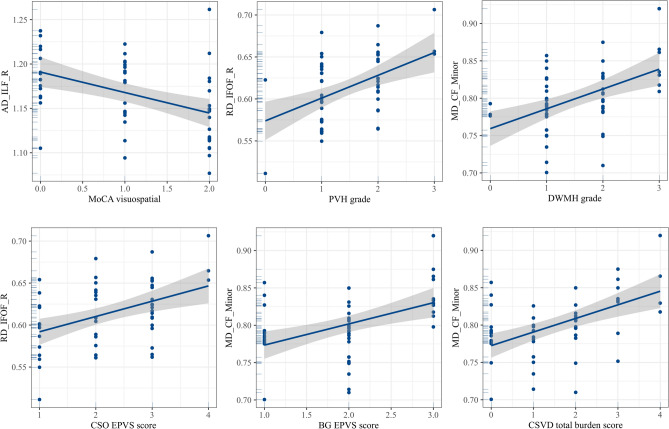
Scatter plots of multiple logistic regression results. *MD* mean diffusivity; *AD* axial diffusivity; *RD* radial diffusivity; *CF* callosum forceps; *IFOF* inferior fronto-occipital fasciculus; *ILF* inferior longitudinal fasciculus; *SLF* superior longitudinal fasciculus; *R* right; *L* left*.*

The multiple regression analysis between DTI metrics and cognitive scores revealed that the mean AD value of right ILF was independent predictor of MoCA visuospatial score (Table 5, Fig. 3).

**Table 5:** Multiple linear regression analysis between DTI metrics and cognitive scores.

	B	β	t	*p*
MoCA visuospatial score				
Mean AD value of right ILF	− 4.627	− 0.257	− 2.220	0.031*

*DTI* diffusion tensor images; *MoCA* Montreal cognitive assessment; *AD* axial diffusivity; *ILF* inferior longitudinal fasciculus.

*p < 0.05.

The multiple regression analysis between DTI metrics and CSVD scores revealed that the mean MD value of right IFOF was independent predictor of PVH grade, CSO-EPVS grade and CSVD total burden score, mean MD value of CF_Minor was independent predictor of DWMH grade, BG-EPVS grade and CSVD total burden score, mean AD value of right ILF was independent predictor of CSO-EPVS grade (Table 6, Fig. 3).

**Table 6:** Multiple linear regression analysis between DTI metrics and CSVD scores.

	B	β	t	*p*
PVH grade				
Mean RD value of right IFOF	7.155	0.417	3.636	0.001*
DWMH grade				
Mean MD value of CF_Minor	7.734	0.425	3.6221	0.001*
CSO-EPVS grade				
Mean RD value of right IFOF	8.534	0.354	2.987	0.004*
BG-EPVS grade				
Mean MD value of CF_Minor	6.414	0.406	3.420	0.001*
CSVD total burden score				
Mean MD value of CF_Minor	8.417	0.292	2.244	0.029*

*DTI* diffusion tensor images; *CSVD* cerebral small vessel disease; *T2DM* type 2 diabetes mellitus; *HC* healthy control; *WMH* white matter hyperintensity; *PVH* periventricular hyperintensity; *DWMH* deep white matter hyperintensity; *EPVS* enlarged perivascular space; *CSO* central semi oval; *BG* basal ganglia; *MD* mean diffusivity; *RD* radial diffusivity; *CF* callosum forceps; *IFOF* inferior fronto-occipital fasciculus.

*p < 0.05.

## Discussion

This study used diffusion metrics, including FA, MD, RD, and AD, to assess the white matter fiber tracts microstructure alterations in T2DM patients. In this study, compared with the HCs, there was extensive damage to the microstructure of the association fibers and CF in T2DM patients, consistent with previous study^37^. Hyperglycemia and broad impairment of microvascular function in T2DM patients will contribute to inflammatory and hypoperfusion, resulting in insufficient brain blood supply^38,39^. Brain is very sensitive to ischemia which could damage white matter fibers microstructural integrity^40^.

In our study, compared with HCs, T2DM patients showed an extensive increase in the mean MD, AD, and RD values of association fibers and CF. Specifically, the MD values of CF_Major, CF_Minor, right IFOF, and right ILF increased, consistent with previous studies^32,41,42^ increased. In the node-wise comparison, the nodes 11–16 of MD in right IFOF in T2DM patients were increased. MD is independent of direction, quantifying the diffusion in extent, and it can sensitively reflect the cell number, edema, and necrosis^43^. Indicating that the number cells of CF_Major, CF_Minor, right IFOF, and right ILF in T2DM patients is reduced, particularly the posterior part of the IFOF. Previous studies^44^ suggest that in the DTI framework, MD account for the diffusion along all three axes of the diffusion tensor with equally weight, i.e. the average value. Therefore, MD is theoretically more robust in evaluating complex fibers, may be the best DTI metrics. In the current study, the AD values of CF_Major, right IFOF, right ILF, and right SLF of T2DM patients were increased compared to the HCs. In the node-wise comparison, the nodes 86–100 of AD in right SLF in T2DM patients were increased. Suggest that the alteration of AD value is mainly located in the posterior part of SLF. The AD value alterations of white matter fiber tracts in T2DM patients in this study were partially consistent with previous research findings^45,46^. However, other studies report opposite results, namely AD values in T2DM patients are decreased for CF_Major, right IFOF and right SLF^32,42,47^. It is suggested that AD changes are more subtle. AD reflects the diffusivity parallel to the white matter fibers, which reflects axon integrity^46,48^. Chronic ischemia and hypoxia caused by chronic hyperglycemia result in axonal loss and fiber degeneration, affecting the distribution of water molecules, expansion of extracellular space^46,49^, leading to an increase in AD. Moreover, our research has also found that the RD values of CF_Minor and bilateral IFOF of T2DM patients were increased compared to the HCs. RD reflects diffusivity perpendicular to axonal fibers, and increased RD is associated with the disruption of the myelin sheath. The IFOF connects the extensive cortex of the frontal, temporal, parietal, and occipital lobes through the external capsule^50^, and the particularity of this anatomical site makes it more vulnerable to be damaged than other fibers. Uniformly increased MD, AD, and RD may reflect a decline in tissue structures that normally prevent the free diffusion of fluid and ensuing changes in fluid volume^51^. However, in this study, no significant differences were found in the FA values between groups. FA has long been one of the most commonly reported quantitative diffusion indicators, usually considered as the most sensitive DTI metrics. Previous studies^15,32–34^ have been reported the white matter FA value abnormalities in patients with T2DM, mainly focusing on corpus callosum, IFOF, SLF and ILF. Nevertheless, there is some evidence supporting the opposite, suggesting that MD and RD values are more sensitive than FA in discovering the white matter microstructure vulnerability^52,53^. This might be due to FA is a relative measure, the quantitative information might be inaccurate in the presence of crossing fibers^44^. However, cerebral white matter voxels containing complex crossing fibers, kissing fibers etc. Therefore, we should be exercise caution when interpreting FA value.

The multiple regression analysis revealed that the mean AD value of ILF_R was independent predictor of MoCA visuospatial score, indicating that axonal loss and fiber degeneration of the right ILF leads to cognitive impairment in T2DM patients, particularly in terms of visuospatial function. The ILF is a fiber connecting the temporal and occipital lobes, closely related to visual and cognitive functions. This is consistent with the results of Gao et al.^54^, i.e. T2DM impairs cognition by attacking ILF. The mean RD value of right IFOF was independent predictor of PVH grade and CSO-EPVS grade, mean MD value of CF_Minor was independent predictor of DWMH grade, BG-EPVS grade and CSVD total burden score, reflected that the damage of CF_Minor and right IFOF would lead to CSVD burden increased in T2DM patients.

This study also has some limitations: (1) the sample size of this study is relatively small, and the participants are recruited from volunteers, which may be biased to some extent; (2) the confounding factors have not been fully accounted for, gut microbiota^55^, age at T2DM onset^56^, lifestyle^57,58^, and treatment^59^ can also have an effect on the cognition of T2DM. The control ability of these confounding factors is insufficient, and more detailed research should be conducted in the future; (3) the CSVD scores are still subjective. In the future, it should be combined with artificial intelligence technology to quantify the burden of CSVD more objectively. (4) some fiber bundles (CH and AF) were adjacent to gray matter and cannot be entirely quantified by AFQ, we cannot know their microstructure changes;(5) due to the cerebral white matter voxel contains multiple fiber populations and complex fiber geometries, DTI metrics should be cautiously interpreted, as they are indirect measures of real biological structures. We would encourage future studies to use other MRI approach that are more robust to the presence of crossing fibers such as diffusion spectrum imaging (DSI)^60^, q-space imaging (QSI)^61^, high-angular-resolution diffusion imaging (HARDI)^62^, and diffusion kurtosis imaging (DKI)^63^, etc.

To conclusion, there is extensive white matter fiber tracts damage in T2DM patients, mainly the damage to commissural and association fibers. It reveals that the mechanism of visuospatial function decline in T2DM patients is related to the deterioration of right ILF, and the damage of CF_Minor and right IFOF is closely related to the CSVD burden increase, which provides a new idea for further understanding the mechanism of cognitive function decline and CSVD burden increase in T2DM.

### Supplementary Information

Supplementary Information.

## Data Availability

All data included in this study are available upon request by contact with the corresponding author.
