# Supplementary material for: White matter microstructure alterations in type 2 diabetes mellitus and its correlation with cerebral small vessel disease and cognitive performance
Source: Sci Rep. 2024 Jan 2;14:270. doi: 10.1038/s41598-023-50768-z (PMC10762026; doi:10.1038/s41598-023-50768-z)
Supplement: Supplementary file 1 — Supplementary Information. [file 41598_2023_50768_MOESM1_ESM.docx]

**Supplementary materials**

**Definition of CSVD MRI feature**

**White matter hyperintensity (WMH):** Hyperintense on T2WI and can appear as isointense or hypointense on T1WI.

**Enlarge perivascular space (EPVS)**: Fluid-filled spaces follow the typical course of a vessel as it goes through grey or white matter.

**Lacune:** A round or ovoid, subcortical, fluid-filled (similar signal as CSF) cavity, 3-15 mm in diameter, consistent with a previous acute small deep brain infarct or haemorrhage in the territory of one perforating arteriole.

**Cerebral microbleed (CMB):** Small areas (2–5 mm in diameter, up to 10 mm) of signal void with associated blooming seen on T2*-weighted or other sequences that are sensitive to susceptibility effects and are generally not seen on CT, or on FLAIR, T1WI, or T2WI.

**Table S1** CSVD MRI feature quantitative

| MRI feature | Quantitative | | | CSVD total burden definition | CSVD total burden score |
| --- | --- | --- | --- | --- | --- |
| WMH | PVH grade | 0 | Absence | ≥DWMH grade 2 or ≥PVH grade 3 | 1 point |
|  |  | 1 | “Caps” or pencil-thin lining |  |  |
|  |  | 2 | Smooth “halo” |  |  |
|  |  | 3 | Irregular PVH extending into the deep white matter |  |  |
|  | DWMH grade | 0 | Absence |  |  |
|  |  | 1 | Punctuate foci |  |  |
|  |  | 2 | Beginning confluence of foci |  |  |
|  |  | 3 | Large confluent areas |  |  |
| EPVS | CSO and BG EPVS score | 0 | Absence | BG-EPVS score 2-4 points | 1 point |
|  |  | 1 | 1-10 |  |  |
|  |  | 2 | 11-20 |  |  |
|  |  | 3 | 21-40 |  |  |
|  |  | 4 | ＞40 |  |  |
| Lacune | Lacune numbers | | | ≥1 lacune | 1 point |
| CMB | CMB numbers | | | ≥1 CMBs | 1 point |

Table S1 Sample size estimation results

|  |  | μ1 | μ2 | δ | σ1 | σ2 | N1 | N2 | N |
| --- | --- | --- | --- | --- | --- | --- | --- | --- | --- |
| FA | IFOF_L | 0.4 | 0.4 | 0 | 0 | 0 | 10 | 10 | 20 |
|  | IFOF_R | 0.4 | 0.4 | 0 | 0 | 0 | 20 | 20 | 40 |
|  | CF_Major | 0.6 | 0.6 | 0 | 0 | 0 | 13 | 13 | 26 |
|  | CF_Minor | 0.5 | 0.5 | 0 | 0 | 0 | 8 | 8 | 16 |
| MD | IFOF_L | 0.8 | 0.9 | -0.1 | 0 | 0.1 | 8 | 8 | 16 |
|  | IFOF_R | 0.8 | 0.9 | -0.1 | 0 | 0 | 4 | 4 | 8 |
|  | CF_Major | 0.8 | 0.9 | -0.1 | 0 | 0.1 | 12 | 12 | 24 |
|  | CF_Minor | 0.8 | 0.9 | -0.1 | 0 | 0 | 5 | 5 | 10 |
| AD | IFOF_L | 1.2 | 1.3 | -0.1 | 0 | 0.1 | 17 | 17 | 34 |
|  | IFOF_R | 1.2 | 1.3 | -0.1 | 0 | 0.1 | 12 | 12 | 24 |
|  | CF_Major | 1.5 | 1.6 | -0.1 | 0 | 0.1 | 13 | 13 | 26 |
|  | CF_Minor | 1.3 | 1.3 | -0.1 | 0 | 0.1 | 7 | 7 | 14 |
| RD | IFOF_L | 0.6 | 0.7 | -0.1 | 0 | 0 | 6 | 6 | 12 |
|  | IFOF_R | 0.6 | 0.7 | -0.1 | 0 | 0 | 4 | 4 | 8 |
|  | CF_Major | 0.5 | 0.6 | -0.1 | 0 | 0.1 | 13 | 13 | 26 |
|  | CF_Minor | 0.5 | 0.6 | -0.1 | 0 | 0.1 | 5 | 5 | 10 |

*FA fractional anisotropy,* *MD mean diffusivity, AD axial diffusivity, RD radial diffusivity, CF callosum forceps, IFOF inferior fronto-occipital fasciculus, R right, L left.*
